# Thermophilic Adaptation of a Minimal Hammerhead Ribozyme Comes with a Functional Penalty

**DOI:** 10.1021/acs.biochem.6c00551

**Published:** 2026-10-19

**Authors:** Ahmed H. Zegher, Samina F. Hanif, Evan M. Kielbasinski, Claire R. Patterson, Carrie Baker, Rigel P. Hall, Kathleen A. Leamy

**Affiliations:** † Department of Chemistry and Biochemistry, Siena University, Loudonville, New York 12211, United States; ‡ Department of Chemistry and Biochemistry, 7447 Gonzaga University, Spokane, Washington 99258, United States

## Abstract

Life has adapted to extreme environments, such as elevated pH, extreme temperatures, and environments with a lack of oxygen. The biomolecules made by organisms that live in extreme environments must adapt to remain folded and functional. Recent computational studies have shown that rRNA, tRNA, and certain mRNAs adapt in thermophilic organisms through an increased GC content. However, few previous studies have assessed the efficiency of adapted thermophilic RNAs. The work presented herein uses minimal hammerhead ribozymes as a model system to determine the effects of high-temperature adaptation, through increased GC base pairing in the ribozyme secondary structure. Two hammerhead ribozymes were designed based upon the minimal hammerhead 16 (HH16) ribozyme, a well-characterized model system. The mesophilic ribozyme has a moderate GC content (44%) in stems I, II, and III, while the thermophilic ribozyme has nearly double the GC content (76%) in those same stems. Both ribozymes share a conserved catalytic core and loop. Thermal denaturation experiments show that the model thermophilic ribozyme is more thermostable with a higher single (cooperative) melting temperature than the model mesophilic ribozyme. However, thermostability is accompanied by a functional penalty. While at certain temperatures, the thermophilic ribozyme reaches the lower observed rates of the mesophilic ribozyme, the thermophilic ribozyme never reaches the same maximal rate of catalysis as the mesophilic ribozyme.

## Graphical abstract

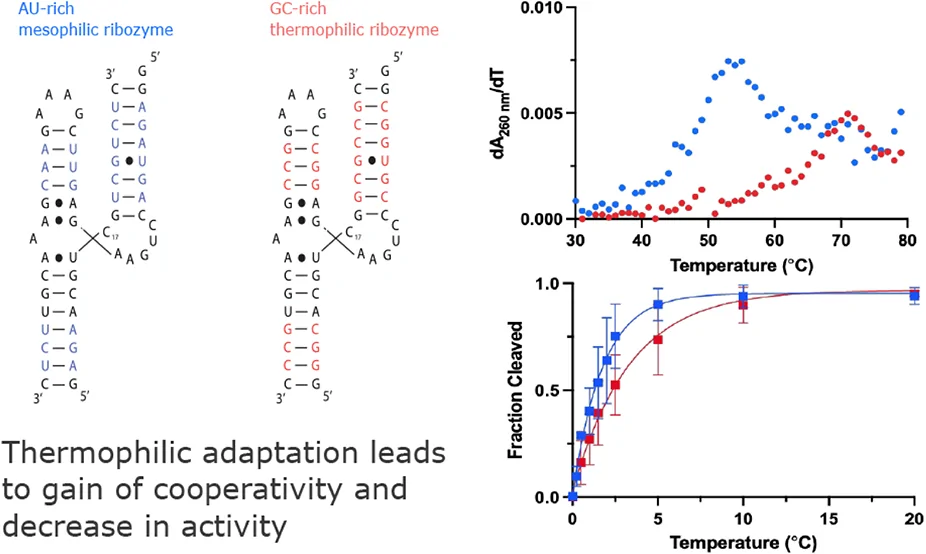

## Introduction

Organisms have adapted to live in a range of temperatures, including the range where water should be frozen or vapor, from psychrophiles that can survive at −20 °C to pyrrothermophiles that survive at 110 °C.^1,2^ For life to survive in these stressed environments, biological molecules have adapted to maintain the structure and function in extreme temperatures. In the thermophilic protein community, a significant amount of work has been done to understand how proteins adapt to remain folded at elevated temperatures.^3–5^ Structural studies have shown that modes of thermophilic protein adaptation largely increase the strength and number of contacts in the protein, which include increasing numbers of hydrophobic residues and contacts in the protein core, ionic interactions, and disulfide bonds.^5^ These types of adaptations strengthen the structure so that it can resist thermal, enzymatic, and chemical denaturation, while maintaining function.^6,7^ Enzymatic studies have revealed that thermophilic protein enzymes often display zero to minimal activity at moderate temperatures, including the temperature ranges at which their mesophilic homologues are the most active.^4^ However, as the temperature increases close to the growth temperature of thermophilic organisms, the activity of thermophilic protein enzymes increases and often reaches the same maximal rate as the mesophilic homologue.^4^

Surprisingly, few studies have been done to understand how functional RNAs, RNAs that adapt a folded structure and perform essential functions in cells, adapt in thermophiles. These types of RNAs are highly conserved and essential for survival and include thermophilic RNA enzymes (ribozymes), riboswitches, tRNAs, and rRNA, among others.^8^ Computational studies have suggested that one potential method of adaptation for these RNAs is an overall increase in sequence GC content.^9–13^ Sequence analysis showed that tRNA and rRNA derived from thermophiles have a higher GC content than the same RNAs derived from mesophiles. Yet, total mRNA, an RNA that tends to exhibit less structure than tRNA and rRNA, does not show an increase in GC content. These works show a general trend into how sequences have adapted in thermophiles but not which regions on the RNAs are adaptable.

Interestingly, a recent study showed that mRNAs encoding the gene malate dehydrogenase in marine mollusks have increasing GC content as mollusks’ adaptation temperature increases, which results in more negative ΔG_fold_ values for predicted regions of mRNA secondary structures.^14^ In addition, previous work has shown that tRNA^Phe^ adapts in thermophiles by increasing GC content specifically in regions of simple secondary structure, while nucleobases involved in tertiary interactions are highly conserved.^15^ In the catalytic domain of RNase P and in tRNA^Phe^, increasing the GC content in secondary structures contributed to thermostability and an increase in cooperativity.^15,16^ Recent studies on a ligand-sensing RNA, the preQ1 riboswitch, showed that a thermophile riboswitch is more stable at higher temperatures and more resistant to urea-induced denaturation than a mesophilic homologue and that the thermophile riboswitch has an unusual protonated cytidine to establish an additional hydrogen bond in the tertiary structure.^17,18^ These studies lead to a general trend in how thermophilic RNAs adapt to extreme temperatures through increases in GC content and show that in certain classes of RNAs, adaptation leads to a more thermostable structure. However, previous work has not investigated the effects of thermal adaptation on self-catalytic RNA function.

In this work, we characterize model mesophilic and thermophilic minimal hammerhead ribozyme constructs to determine how increasing the GC content in regions of secondary structure affects thermal stability and the ability to perform self-cleavage. The model ribozymes were designed on the basis of the minimal hammerhead 16 (HH16) ribozyme by Uhlenbeck and colleagues. This system was selected because it is a well-characterized ribozyme that does not form stable alternative structures and has a faster rate of substrate dissociation than the rate of cleavage.^19,20^ This is critical because thermostability is about the functional state, the state with a tertiary structure, and ribozymes are ideal for probing this state because they reveal it through bond cleavage.^21,22^

Our findings indicate that an increase in the number of GC base pairs in the three stems of the HH16 ribozyme does significantly increase the thermal stability of the ribozyme, allowing it to maintain its function in the temperature range at which thermophilic organisms live. However, this increase in thermal stability comes with a functional penalty. At certain temperatures, the thermophilic ribozyme reaches the lowest observed rates of the mesophilic ribozyme; however, the maximal rate of catalysis is slower for the thermophilic ribozyme than that for the mesophilic ribozyme. Our results show that increasing GC content in stem regions is a plausible mode for high-temperature adaptation in thermophiles but that adaptation comes with a functional penalty.

## Materials and Methods

### Materials

Hammerhead substrate oligos were purchased with a 5′ carboxyfluorescein label from Integrated DNA Technologies (IDT, Coralville, IA) with high-pressure liquid chromatography purification. The T7 promoter sequence and template DNA oligos used for the transcription of hammerhead enzymes were purchased from IDT and used without further purification. T7 polymerase was purchased from New England Biolabs. The chemicals were purchased from Sigma-Aldrich.

### RNA Sequences for Kinetics and Preparation

The hammerhead ribozyme enzyme and substrate sequences used in this study are listed below. The sequences are based on the minimal hammerhead 16 ribozyme designed by Uhlenbeck and coworkers that has no stable alternative structures and a fast rate of cleavage and substrate dissociation due to the 16 strong base pairs between the enzyme and the substrate.^19,20^ Seven base pairs in the stems were changed so that the mesophilic ribozyme has 44% GC content (50%, 33%, and 50% GC content in stems I, II, and III, respectively), and the thermophilic ribozyme has 76% GC content (87.5%, 66%, and 75% GC content in stems I, II, and III, respectively). Bases in the catalytic core and loops were not modified from the ribozyme designed by Uhlenbeck and colleagues (Figure 1).

**Figure 1:**
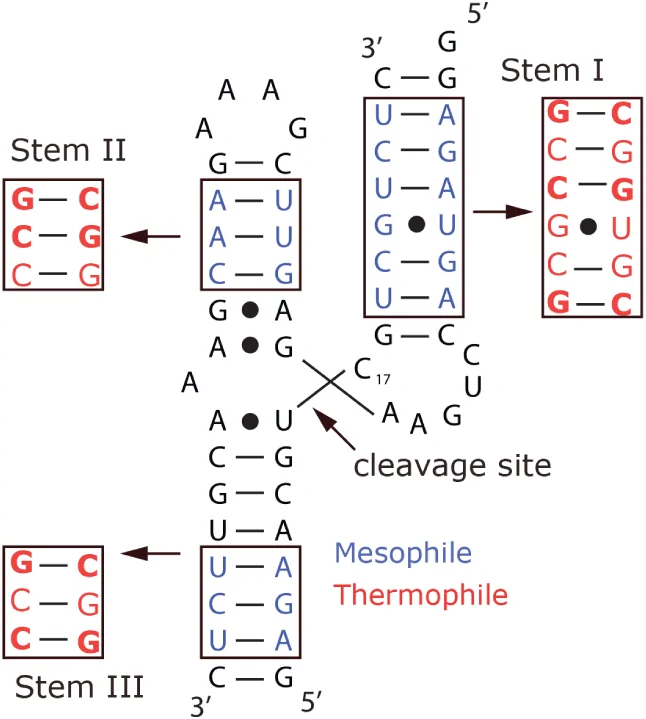
Model mesophilic and thermophilic minimal hammerhead ribozyme constructs. The mesophilic construct contains the black and blue nucleotides, and the thermophilic construct contains increased GC content in stems I, II, and III, shown by the red bolded nucleotides in the boxed regions. The cleavage site is indicated with an arrow.

RNAStructure was used to predict the overall structure and free energy of both constructs and found a single stable structure for the mesophilic ribozyme and two stable structures for the thermophilic ribozyme that differ by one base-pairing interaction in stem I (Figure S1). These structures are very similar to the structure of the minimal hammerhead 16 ribozyme, and minor differences between the predicted structures are shown in Figure S1.

**Mesophilic enzyme:** 5′GGAGAUGACCUGAAGAGUUCGAAAGAACGAAACGUUCUC

**Thermophilic enzyme:** 5′GGCGGUGCCCUGAAGAGGCCGAAAGGCCGAAACGUGCCC

**Mesophilic substrate:** 5′FAM-GAGAACGUCGUCGUCUC

**Thermophilic substrate:** 5′FAM-GGGCACGUCGGCGCCGC

**T7 promoter DNA:** 5′d­(TAATACGACTCACTATA)

Enzyme RNAs were transcribed by *in vitro* T7 polymerase transcription and all sequences start with Gs to facilitate transcription.^23^ Template DNA, T7 promoter DNA, and sodium chloride were combined with 10 mM Tris/1 mM ethylenediaminetetraacetic acid (EDTA) buffer at pH 7.0 (1X TE buffer) and were annealed by heating at 95 °C for 3 min and then cooling at room temperature for 10 min. After annealing, T7 polymerase enzyme and 10X transcription mix (containing 400 mM Tris buffer, pH 8.0, 20 mM dithiothreitol, 10 mM spermidine, 250 mM magnesium chloride, and 50 mM nucleotide triphosphates) were added to the annealed DNA. The solution was carefully mixed and incubated at 37 °C for 4 h. Transcription reactions to make the thermophilic enzyme contained an additional 10% dimethyl sulfoxide to improve the yields of the GC-rich transcription.^24^ Transcription reactions were quenched by adding 2X formamide loading dye containing 50 mM EDTA and frozen overnight at −20 °C or immediately gel-purified by 10% polyacrylamide gel electrophoresis (PAGE). The RNA was recovered from the gel by a crush and soak procedure followed by an ethanol precipitation, then the RNA was buffer exchanged using an Amicon ultracentrifugal filter (3 kDa molecular weight cutoff) and stored in 1X TE buffer, pH 7.0, at −20 °C.

### Kinetic Assays

For single-turnover kinetic assays, stock solutions of the enzyme strand and substrate strand in 1X TE buffer were combined and renatured by heating at 95 °C for 2 min and then cooling at room temperature for 10 min. The RNA was then combined with a 10X buffer containing sodium cacodylate, pH 7.5, and potassium chloride, and the solution was incubated at the reaction temperature for 5 min to equilibrate. After equilibration, a zero time point was removed and combined with an equal volume of 95% formamide loading dye containing 50 mM EDTA, and the sample was placed directly on ice. To initiate the reaction, an appropriate volume of 10X magnesium chloride was added to the reaction mixture and mixed vigorously, while incubating at the reaction temperature. Final concentrations were 1 μM enzyme, 100 nM substrate, 10 mM sodium cacodylate, pH 7.5, 140 mM potassium chloride, and either 2.0 or 10.0 mM magnesium chloride. Reaction progress was monitored by removal of aliquots of the reaction at the appropriate time points, quenching the reaction with an equal volume of 95% formamide loading dye containing 50 mM EDTA directly on ice. Time points were fractionated via denaturing 15% PAGE. Gels were frozen at −80 °C and visualized using a Bio-Rad ChemiDoc MP imaging system with detection of fluorescein. ImageJ was used to analyze the gels, and plots of fraction cleaved, *f*_*c*_, versus time were fit to a single exponential (eq 1) in Prism 10. All kinetic reactions were performed in duplicate.

1 $$f_{c}=A+Be^{-k_{obs}t}$$

### RNA Substrate Sequences for Native Gels and Thermal Denaturation

Removing the 2’-hydroxyl on the C17 ribose sugar prevents catalysis,^25^ so substrates with a deoxyribose sugar on C17 were used for native gels and thermal denaturation experiments to prevent catalysis from occurring during the experiment.

**Deoxy mesophilic substrate:** 5′GAGAACGUd­(C)­GUCGUCUC

**Deoxy thermophilic substrate:** 5′GGGCACGUd­(C)­GGCGCCGC

### Native Gels

Samples were run on a nondenaturing 10% acrylamide (29:1 acrylamide:bis­(acrylamide)) native gel, with a buffer composed of 34 mM Tris base, 660 mM HEPES acid, 1 mM EDTA, and either 2.0 or 10.0 mM MgCl_2_ in the gel. RNA samples were prepared as described for kinetics experiments, but with unlabeled deoxy substrates and with final concentrations of 1 μM annealed ribozyme to 1 μM deoxy substrate. Gels were run at 5 W at room temperature with a temperature probe attached to the outside of the gel plate to ensure the gel did not overheat. Gels were stained with a SYBR Gold stain for 5 min and scanned with a Bio-Rad ChemiDoc MP imaging system.^25^

### UV-Detected Thermal Denaturation

Enzyme and unlabeled deoxy substrate were renatured by heating at 95 °C for 2 min in the presence of 10X buffer containing sodium cacodylate, pH 7.5, and potassium chloride. The samples were then cooled at room temperature for 10 min. After cooling, magnesium chloride was added and the sample was incubated at room temperature for 10 min. Final concentrations were 1.5 μM enzyme, 1.5 μM substrate, 10 mM sodium cacodylate, pH 7.5, 140 mM potassium chloride, and 2.0 or 10.0 mM magnesium chloride. Samples were spun at 4 °C for 5 min to remove bubbles before being pipetted into the cuvette. Experiments were performed on an Agilent Cary 100 UV–vis spectrophotometer with an external water bath to regulate temperature and an external temperature probe in the sample to monitor sample temperature. The renatured sample was heated from 30 to 90 °C, at a rate of 1.0 °C/min, with data collected at 260 nm every 1.0 °C. Each experiment was performed in duplicate and the data presented is an average of both trials.

### UV-Detected Thermal Denaturation Data Analysis

In an effort to obtain thermodynamic parameters, all melting data sets were fit to a 2-state unfolding model (eq 2) in Python.

2 $$A\left(T\right)=\frac{\left(m_{U}T+b_{U}\right)+\left(m_{F}T+b_{F}\right)e^{\frac{\Delta H^{{}^{\circ}}}{R}\left(\frac{1}{T_{M}+273.15}-\frac{1}{T+273.15}\right)}}{1+e^{\frac{\Delta H^{{}^{\circ}}}{R}\left(\frac{1}{T_{M}+273.15}-\frac{1}{T+273.15}\right)}}$$

For a two-state model, the RNA is at equilibrium between folded, *F*, and unfolded, *U*, (eq 3) without a population of intermediate states that can be described by the equilibrium constant, *K*, (eq 4) or the fraction unfolded (*f*_*U*_) (eq 5).

3 F⇌U

4 $$K=\frac{\left[F\right]}{\left[U\right]}$$

5 $$f_{U}=\frac{\left[U\right]}{\left[U\right]+\left[F\right]}$$

The absorbance at each temperature (eq 6) comes from both the absorbance of the folded state (eq 7) and the absorbance of the unfolded state (eq 8) and is weighted by the fraction unfolded. The absorbance of the folded state can be defined by the y-intercept, *b_F_*, and the slope, *m_F_*, of the linear lower baseline (eq 7). The absorbance of the unfolded baseline can be defined in a similar manner, where *b_U_* is the y-intercept of the linear upper baseline and *m_U_* is the slope of the linear upper baseline (eq 8).

6 $$A\left(T\right)=A_{U}f_{U}+A_{F}f_{F}$$

7 $$A_{F}\left(T\right)=m_{F}T+b_{F}$$

8 $$A_{U}\left(T\right)=m_{U}T+b_{U}$$

Normalize relative to the unfolded state, *U*, and divide *f*_*U*_ by [U] and then substitute in the equilibrium constant to get *f*_*U*_ as a function of *K* (eq 9). The equilibrium constant changes with temperature, and using the van’t Hoff equation, we can write *K* in terms of the ideal gas constant, *R*, and the enthalpy of unfolding, Δ*H*° (eq 10):

9 $$f_{U}=\frac{1}{1+K}$$

10 $$\frac{d\ln\left(K\right)}{d\left(\frac{1}{T}\right)}=\frac{-\Delta H^{{}^{\circ}}}{R}$$

eq 10 can be integrated from 1/*T_M_* to 1/*T*, where *K* = 1 at the melting temperature, *T_M_*, where the populations of *F* and *U* are equal (eq 11 and eq 12).

11 $$\ln\left(K\right)=\frac{\Delta H^{{}^{\circ}}}{R}\left(\frac{1}{T_{M}}-\frac{1}{T}\right)$$

12 $$K=e^{\frac{\Delta H^{{}^{\circ}}}{R}\left(\frac{1}{T_{M}}-\frac{1}{T}\right)}$$

To fit the melting data (eq 2), the above derived equations are substituted into eq 6, where *R* is the ideal gas constant, 0.001987 kcal K^–1^ mol^–1^.

## Results and Discussion

### Design of Model Mesophilic and Thermophilic Hammerhead Ribozymes

The hammerhead ribozyme constructs used in this study were based upon the minimal hammerhead 16 construct designed by Uhlenbeck and co-workers^26^ with modifications to the stem base pairing to generate an AU-rich construct, referred to as the “mesophilic ribozyme” and a GC-rich construct, referred to as the “thermophilic ribozyme” (Figure 1). This minimal hammerhead 16 ribozyme is well-characterized and does not have alternative stable structures.^27,28^ Bases in regions of the RNA that are known to form tertiary interactions, are part of the catalytic core,^27^ or have been suggested to be necessary for function were not changed (Figure 1). The sequence of the thermophilic ribozyme was input into QGRS Mapper^29^ to determine if the sequence is likely to form a G-quadruplex due to the high GC content, and no stable G-quadruplex structures were predicted. RNAStructure^30^ was used to predict the free energy of both ribozyme biomolecular structures and to determine whether the sequences were predicted to form the correct secondary structures. RNAStructure predicts that the mesophilic ribozyme forms one stable structure and that the thermophilic ribozyme forms two stable structures. All three predicted ribozyme structures have the secondary contacts in Figure 1, except for the two G·A mismatches at the end of stem II (Figure S1). In each predicted structure, C17, the catalytic residue, and the residues in the catalytic core are single-stranded. The thermophilic ribozyme is predicted to form two similar structures that differ by one base-pairing interaction in stem I and differ by a free energy of only −1.9 kcal/mol (Figure S1). RNAStructure predicted that the thermophilic ribozyme is significantly more stable than the mesophilic ribozyme, with free energies of folding of −38.2 and −24.0 kcal/mol, respectively, giving an overall increase in stability for the thermophilic ribozyme of −14.2 kcal/mol just from increasing the GC content by seven base pairs in the stems (Figure 1). Since the interactions not predicted by RNAStructure are GA mismatches at the end of a stem, which are difficult to predict and contribute minimally to stability,^31,32^ we consider this validation that the designed ribozymes adapt the minimal hammerhead fold.

### Increased Base-Pairing Strength Stabilizes the Thermophilic Ribozyme

In prokaryotic cells, the free Mg^2+^ concentration is approximately 2.0–3.0 mM, and in eukaryotic cells, the free Mg^2+^ concentration is slightly lower at 0.5–1.0 mM.^33–37^ As most known hammerhead ribozymes are found in prokaryotic organisms, the cleavage of mesophilic and thermophilic hammerhead ribozymes was studied in prokaryotic cellular ionic conditions, of 140 mM K^+34^ and 2.0 mM Mg^2+^. The activity of each ribozyme was determined at 30 °C, and at increasing temperatures of 5 °C, until a temperature at which the ribozyme was no longer active.

In 2.0 mM Mg^2+^, the mesophilic ribozyme undergoes rapid self-cleavage between 30 and 50 °C, with observed rate constants between 0.0116 and 0.033 min^–1^ (Table 1, Figure 2).

**Table 1:** Kinetic Parameters for Mesophilic and Thermophilic Ribozymes in 2.0 and 10.0 mM Mg^a^ ^2+^

	Mesophilic Ribozyme				Thermophilic Ribozyme			
	2.0 mM Mg^2+^		10.0 mM Mg^2+^		2.0 mM Mg^2+^		10.0 mM Mg^2+^	
Temperature (°C)	k_obs_ (min^–1^)	Std Dev	k_obs_ (min^–1^)	Std Dev	k_obs_ (min^–1^)	Std Dev	k_obs_ (min^–1^)	Std Dev
**30**	0.026	0.002	0.16	0.03	0.005	0.002	0.024	0.005
**35**	0.020	0.002	0.097	0.003	0.010	0.001	0.091	0.007
**40**	0.033	0.003	0.52	0.05	0.010	0.001	0.071	0.006
**45**	0.015	0.002	0.30	0.02	0.008	0.002	0.09	0.01
**50**	0.0116	0.0007	0.56	0.06	0.022	0.001	0.095	0.006
**55**	0.005	0.001	0.57	0.07	0.009	0.002	0.63	0.06
**60**	ND	ND	0.78	0.06	0.008	0.001	0.162	0.009
**65**	ND	ND	1.60	0.30	0.013	0.004	0.30	0.04
**70**	ND	ND	ND	ND	ND	ND	0.12	0.02
**75**	ND	ND	ND	ND	ND	ND	0.16	0.01
**80**	ND	ND	ND	ND	ND	ND	0.056	0.009

a ND indicates temperatures at which rate constants could not be determined because the ribozyme is either not active or shows signs of degradation.

**Figure 2:**
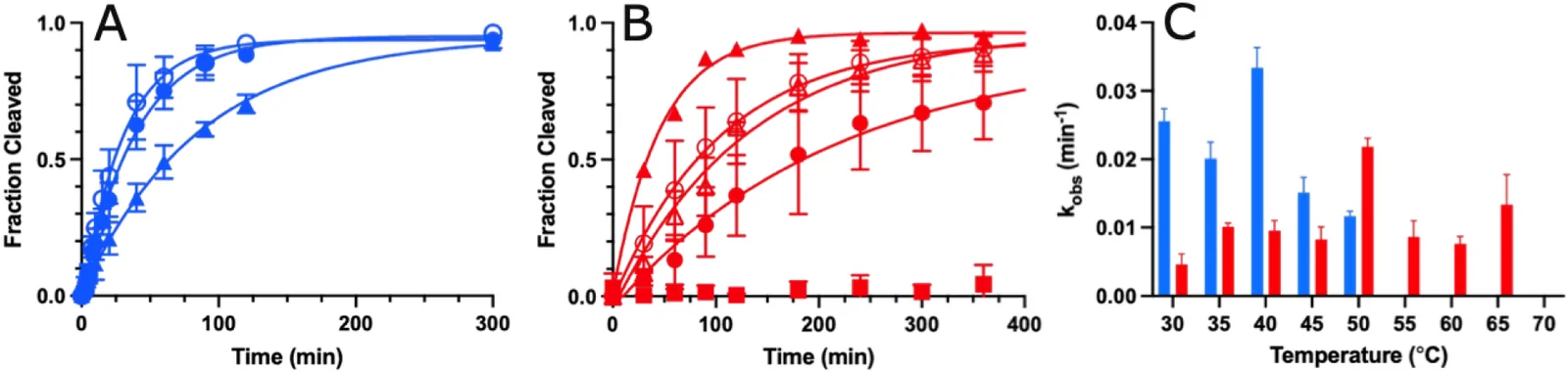
Kinetic profiles of the mesophilic and thermophilic ribozymes in 2.0 mM Mg^2+^. Fraction cleaved as a function of time for (A) mesophilic (blue) and (B) thermophilic (red) ribozymes in 2.0 mM Mg^2+^ at 30 °C (closed circles), 40 °C (open circles), 50 °C (closed triangles), 60 °C (open triangles), and 70 °C (closed squares). The fraction cleaved data was fit to obtain (C) k_obs_ for the mesophilic (blue) and thermophilic (red) ribozymes from 30 to 65 °C, at 5 °C increments, at temperatures where the ribozyme is active. Data presented are the average and standard deviation of two replicates. The rate of the thermophilic ribozyme at 70 °C could not be determined due to the low activity. At 55 °C, the mesophilic ribozyme shows signs of degradation (see Figure S2), and the rate constants for self-cleavage were not determined at temperatures where degradation was observed.

The mesophilic ribozyme peaks in activity at 40 °C with an observed rate constant of 0.033 min^–1^, before showing a very steep decline in activity as temperature increases, until the ribozyme is minimally at 50 °C with an observed rate constant of 0.0116 min^–1^. At 55 °C, gels of the kinetic experiments show substrate cleavage and RNA degradation, suggesting that the mesophilic ribozyme is at least partially unfolded, solvent-accessible, and degrading, so a rate constant was not determined (Figure S2). Mesophilic organisms live at moderate temperatures and grow optimally between 20 and 45 °C, indicating that our mesophilic hammerhead ribozyme construct is a good model system for a mesophilic ribozyme since it displays peak activity at 40 °C and is the most active in the temperature range at which mesophiles live.

The rate constants for thermophilic ribozyme self-cleavage were also determined in ionic conditions that mimic those of prokaryotic cells. In 2.0 mM Mg^2+^, the thermophilic ribozyme is catalytically active from 30 to 65 °C, a higher maximum temperature than the mesophilic ribozyme by 15 °C. Indeed, the thermophilic ribozyme reaches 100% cleavage at elevated temperatures showing that increasing the GC content in regions of secondary structure does increase the stability of the ribozyme functional state, which results in activity at higher temperatures (Table 1, Figure 2). The thermophilic ribozyme peaks in activity at 50 °C with a rate constant of 0.022 min^–1^ and then shows a decline in activity until the ribozyme is inactive at 70 °C. However, this increase in stability comes with a slight functional penalty. The rate constants of the thermophile are similar to the lower observed rate constants for the mesophile; for example, the mesophilic ribozyme cleaves with a rate of 0.0116 min^–1^ at 50 °C and the thermophilic ribozyme cleaves with a rate of 0.013 min^–1^ at 65 °C. However, the thermophilic ribozyme’s maximal observed rate of 0.022 min^–1^ at 50 °C is approximately 1.5-fold slower than the mesophilic ribozyme’s maximal observed rate of 0.033 min^–1^ at 40 °C. While slower, the thermophilic ribozyme does reach 100% cleavage, indicating that the ribozyme population does fold into the native state during the time course of the self-cleavage reaction at more extreme temperatures.

### Higher Concentrations of Mg^2+^ Stabilize the Thermophilic Ribozyme

RNA folding and stability are heavily influenced by the solution environment, particularly by the concentration of free divalent ions,^22,23^ as shown by several studies.^38,39^ Increasing the concentration of divalent ions in solution can result in a large change in the rate of catalysis for ribozymes^40,41^ as divalent ions assist in RNA folding by minimizing repulsions from the phosphate backbone and divalent ions can participate in catalysis. Therefore, it was hypothesized that the thermophilic ribozyme could reach the same maximal rate as the mesophilic ribozyme under conditions of higher concentrations of Mg^2+^, such as 10.0 mM, which is commonly used to study ribozymes.^27,42,43^

In an effort to stabilize the native state, and to determine if increased Mg^2+^ concentrations will enhance the rate of the thermophilic ribozyme to the same level as the mesophilic ribozyme, the activities of the mesophilic and thermophilic ribozymes were investigated in the presence of 10 mM Mg^2^. The magnitude of the maximal rate constant of cleavage of the mesophilic ribozyme increased almost 50-fold in 10 mM Mg^2+^ compared to 2.0 mM Mg^2+^, with a maximal observed rate constant of 1.6 min^–1^ at 65 °C, which is approximately 50-fold faster than the observed mesophilic rate constant of 0.033 min^–1^ at 40 °C in 2.0 mM Mg^2+^ (Table 1, Figure 3). These results clearly show that increased levels of divalent ions assist in ribozyme folding and catalysis, resulting in a more thermostable ribozyme with a faster rate of catalysis.

**Figure 3:**
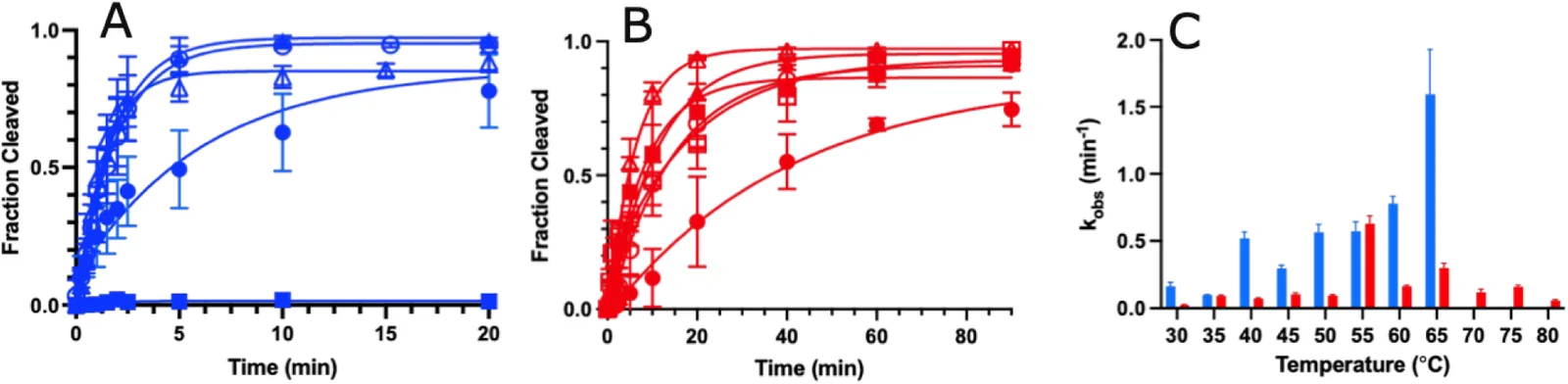
Kinetic profiles of the mesophilic and thermophilic ribozymes in 10.0 mM Mg^2+^. Fraction cleaved as a function of time for (A) mesophilic (blue) and (B) thermophilic (red) ribozymes in 2.0 mM Mg^2+^ at 30 °C (closed circles), 40 °C (open circles), 50 °C (closed triangles), 60 °C (open triangles), 70 °C (closed squares), and 80 °C (open squares). The fraction cleaved data was fit to obtain (C) k_obs_ for the mesophilic (blue) and thermophilic (red) ribozymes from 30 to 80 °C, at 5 °C increments, at temperatures where the ribozyme is active. Data presented are the average and standard deviation of two replicates. The mesophilic ribozyme is inactive at 70 °C and higher, so no rates are provided beyond 65 °C. At temperatures above 85 °C, the thermophilic ribozyme shows signs of degradation (see Figure S2), and rate constants for self-cleavage were not determined at temperatures where degradation was observed.

The thermostability, and the activity, of the thermophilic ribozyme also increased in the presence of higher concentrations of Mg^2+^. The ribozyme is active up to 80 °C (Table 1, Figure 3) with a rate constant of 0.056 min^–1^ at 80 °C and reached a maximal rate constant of 0.63 min^–1^ at 55 °C. At temperatures above 80 °C, analysis of the kinetic gels shows that the thermophilic ribozyme is cleaved at multiple positions and is self-degrading instead of cleaving specifically (Figure S2), precluding analysis. These data again showed that increased concentrations of Mg^2+^ stabilized the native state and increased the thermostability of the thermophilic ribozyme, with a 28-fold change in the maximal rate constant obtained from a rate constant of 0.022 min^–1^ at 50 °C in 2.0 mM Mg^2+^ to 0.63 min^–1^ at 55 °C in 10.0 mM Mg^2+^.

Thermophiles grow optimally between 50 and 85 °C, and the increased stability of the thermophile secondary structure did increase the overall stability of the RNA so that it is active at temperatures that the thermophilic organisms live at. The mesophilic and thermophilic ribozymes self-cleave with very similar rate constants at 55 °C, of 0.57 min^–1^ and 0.63 min^–1^, respectively. While the thermophilic ribozyme is active at higher temperatures in elevated Mg^2+^, and reaches 100% cleavage, the observed maximal rate constants of cleavage for the thermophilic ribozyme are still slower than the mesophilic ribozyme, by approximately 2.5-fold. Similar to the function of the ribozymes at low concentrations of Mg^2+^, increasing the GC content in the secondary structures of the thermophilic ribozyme increased the ribozyme’s thermostability, but again, it seems to have come with a functional penalty. There are a few reasons why the thermophilic ribozyme could be significantly slower than the mesophilic ribozyme: the ribozyme could have a misfolded population that is slow to reach the native state (see potential alternative base pairing in Figure S1) or the ribozyme could be less dynamic due to the increased GC content of the secondary structure.

### Native Gels Show the Ribozyme Forms a More Compact Structure at Elevated Mg^2+^

Native gels were run with both ribozymes at room temperature, in either 2.0 or 10.0 mM Mg^2+^, to determine whether alternative structures could be detected. For native gels, the substrate contained a deoxyribose sugar at the cleavage site to prevent catalysis and to keep the ribozyme population in the annealed precleavage state. In 2.0 mM Mg^2+^, both the thermophilic and mesophilic ribozymes show two bands on the native gel that run close to each other (Figure 4).

**Figure 4:**
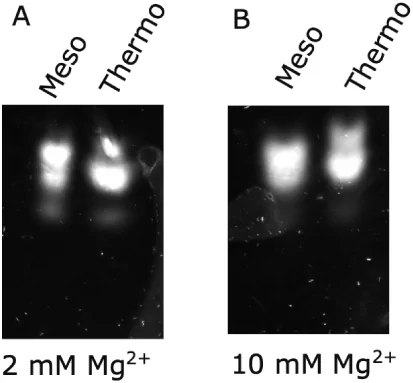
Native gels of the mesophilic and thermophilic ribozymes in (A) 2.0 mM Mg^2+^ and (B) 10.0 mM Mg^2+^.

The faster-mobility band is more populated for the thermophilic ribozyme, suggesting a higher population of the functional state. This indicates that the mesophilic ribozyme has a lower population in the functional state than the thermophilic ribozyme, meaning that the mesophilic ribozyme is self-cleaving faster despite having a lower population in the functional state. On the native gels, there appear to be two bands, and the faster-mobility band is more populated for the thermophilic ribozyme than the mesophilic ribozyme, suggesting a faster intrinsic activity for the mesophilic ribozyme than the thermophilic ribozyme. Similar observations are made in 10.0 mM Mg^2+^ (Figure 4). However, the native gels do not suggest that a larger population of the thermophilic ribozyme is in an alternative structure than the mesophilic ribozyme, therefore, misfolding of the thermophilic ribozyme is not suspected to be a cause for the slow rate.

### Increase in GC Content Leads to a Gain in Folding Cooperativity

Thermal denaturation experiments were performed to determine the melting temperatures of both ribozymes and to test whether multiple unfolding transitions could be detected in the unfolding profile of the thermophilic ribozyme. Heat was used to denature the mesophilic and thermophilic ribozymes in 10 mM sodium cacodylate (pH 7.5) with 140 mM potassium chloride, and additional divalent ions, either 2.0 mM Mg^2+^ or 10.0 mM Mg^2+^, while the absorbance was measured at 260 nm. A substrate with a deoxyribose sugar at the cleavage site was used to prevent the ribozymes from cleaving during the experiment, which could result in multiple stable structures in solution at once, making the data challenging to interpret. We note that the substrate is quite long with 8 base pairs in stem I and 8 base pairs in stem III, compared to 6 base pairs—with 2 GA mismatches in stem II, meaning that the enzyme–substrate base pairing should form a very stable interaction and should not denature before the rest of the ribozyme.

The first derivative curves of the melts show unfolding over a wide temperature range, for the mesophilic ribozyme in both 2.0 and 10.0 mM Mg^2+^. The thermophilic ribozyme appears to unfold in a single, sharp transition, in 2.0 and 10.0 mM Mg^2+^ (Figure 5). These data suggest that the mesophilic ribozyme either unfolds in a multistate manner with tertiary structure unfolding before secondary structure or as native gels suggest that there are multiple folded populations with differing stabilities (Figure 5). The single transition for the thermophilic ribozyme suggests that the increase in stem base-pairing increases the stability of the secondary structure so that the secondary and tertiary interactions unfold cooperatively in a single clear transition. In addition, the cooperative nature of this transition suggests that a single population is favored by the thermophilic ribozyme. Melting curves for both ribozymes show that an increase in the concentration of Mg^2+^ shifts the unfolding transitions to higher temperatures, indicative of a more stable RNA (Figure 5).

**Figure 5:**
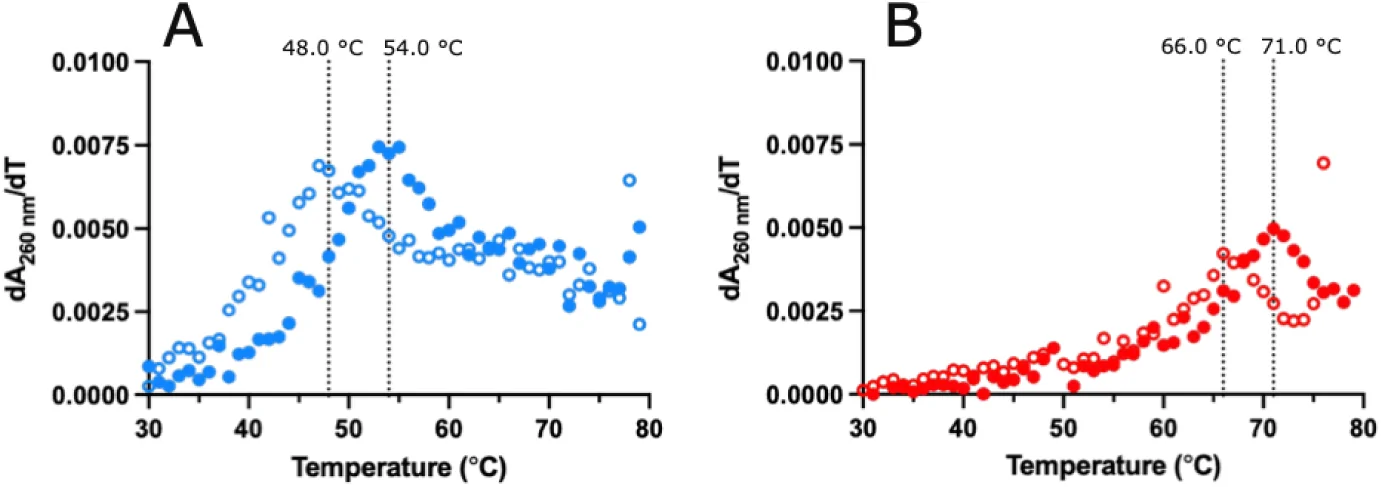
First-derivative curves of UV-detected melting curves of the (A) mesophilic ribozyme and (B) thermophilic ribozyme in 2.0 mM Mg^2+^ (open circles) and 10.0 mM Mg^2+^ (closed circles). Black vertical lines show the lowest-temperature peaks in the first-derivative curves, which are considered to be the unfolding temperatures (Table 2).

The raw melting data was fit to a 2-state model in an effort to obtain melting temperatures (eq 2) and thermodynamic data. Data above 80 °C were not analyzed due to sample evaporation.^44^ The mesophilic ribozyme unfolds at a low temperature, showing the start of unfolding close to 40 °C in both low and high concentrations of Mg^2+^, so there is limited lower baseline data for the mesophilic ribozyme. The thermophilic ribozyme unfolds at a much higher temperature and shows signs of unfolding at 80 °C, where the sample began to evaporate, so there is limited upper baseline data for the thermophilic ribozyme. Due to the lack of baseline data for the ribozymes, the two-state fit was of poor quality (Figure S3), and the thermal denaturation data is discussed in a qualitative manner. We define the “unfolding temperature” as the temperature of the first peak in the first derivative of the denaturation data (Table 2). In all cases, we take the lowest temperature transition as that representing the thermostability of the ribozyme because melting of any structure should lead to loss of activity.

**Table 2:** Unfolding Temperatures of the Mesophilic and Thermophilic Ribozymes

	2.0 mM Mg^2+^	10.0 mM Mg^2+^
**Mesophilic Ribozyme**	48.0	54.0
**Thermophilic Ribozyme**	66.0	71.0

In a biological concentration of 2.0 mM Mg^2+^, the mesophilic ribozyme unfolds over a wide temperature range, with an unfolding temperature of 48.0 °C, indicating that the mesophilic ribozyme is unfolding in a multistate uncooperative manner or that there are multiple stable states. In the same conditions, the thermophilic ribozyme unfolds over a short temperature range, with an unfolding temperature of 66.0 °C. These melting temperatures correlate with the activity observed through kinetics. In low Mg^2+^ conditions, the mesophilic ribozyme was catalytically active at 50 °C, and showed minimal catalytic activity at 55 °C, very close to the unfolding temperature of 48.0 °C. Similarly, in 10.0 mM Mg^2+^, the mesophilic ribozyme shows catalytic activity until 65 °C, correlating with the melting temperature of 54.0 °C over a very broad transition. The mesophilic ribozyme is still active at 65 °C, despite having a *T_M_* 10.5 °C lower since there is still a fraction of the ribozyme that is folded and active and heat increases the dynamics and activity. Denaturation experiments support this, as there is some species of the mesophilic ribozyme that is still unfolding at 65 °C.

The thermophilic ribozyme is catalytically active in 2.0 mM Mg^2+^ until 65 °C and in 10.0 mM Mg^2+^ until 80 °C. The maximum temperatures of catalytic activity of the thermophilic ribozyme correlate directly with the unfolding temperatures, which are 66.0 °C in 2.0 mM Mg^2+^ and 71.0 °C in 10.0 mM Mg^2+^. For both ribozymes in 2.0 and 10.0 mM Mg^2+^, the unfolding temperatures correlate with the loss of catalytic activity. These data suggest that the ribozymes only become inactive at temperatures at which they are unfolded, although in high Mg^2+^ concentrations, the loss of RNA folding at temperatures above the unfolding temperature might be compensated for by gains in the rate of cleavage. The melting data clearly show that the increase in GC content in the thermophilic ribozyme results in a more cooperative folding transition that does not have an observable population of intermediate states.

## Conclusions

Previous studies have shown that thermophiles adapt to extreme heat by increasing the GC content in functional RNAs such as tRNAs and rRNAs.^9–11,13^ However, few studies have examined where those adaptations occur in the RNA structure; in tRNA^Phe^, thermal adaptations occur in regions of simple secondary structures, and tRNA^Phe^ from mesophilic and thermophilic species have the same tertiary contacts,^15^ suggesting that bases involved in tertiary interactions are less adaptable. Our study investigates the effects of an increased GC content in regions of secondary structures on the function of the minimal hammerhead ribozyme. Our results show that increasing the GC content in regions of simple secondary structures in the minimal hammerhead ribozyme increases the overall stability of the natively folded RNA and increases the level of folding cooperativity. RNA folds hierarchically, with the tertiary structure forming after the secondary structure forms, so when the penultimate, secondary structure state is stabilized, the final state also increases in stability. When the penultimate state and final state have similar stabilities, the RNA folds cooperatively without populating intermediate states. Our results show that this increase in stability and cooperativity comes with a slight functional penalty. While at certain temperatures, the thermophilic ribozyme reaches the lower rates observed in the mesophilic ribozyme, the thermophilic ribozyme cleaves at a slower maximal rate constant than the mesophilic ribozyme. Since both ribozymes share the same catalytic core and loops, the slower rate of catalysis for the thermophilic ribozyme must be caused by changes in the GC content of the three stems.

An increase in GC content stabilizes the ribozyme by increasing the strength of the stacking interactions and the number of hydrogen-bonding interactions in the secondary structure. It is likely that this increase in stability results in a decrease in flexibility of the RNA. The greater resistance of the thermophilic RNA to degradation (Figure S2) supports its lower dynamics.^45,46^ Thermophilic protein enzymes tend to be barely active at room temperatures; however, most reach the same level of activity as their mesophilic homologue at their optimal growth temperature.^4^ It is possible that the slower rate of the thermophilic ribozyme comes from the increase in base stacking and hydrogen bonding, causing the enzyme to lack the flexibility needed for maximal function. Indeed, the ribozyme reaction rates increased at higher temperatures until reaching the melting temperature. Heat provides energy for dynamics, which can explain the increased reaction rate.

The minimal hammerhead ribozyme constructs used in this study were transcribed *in vitro* with T7 polymerase using unmodified nucleotide triphosphates. In nature, RNAs are heavily modified, and there are over 130 known RNA modifications that are naturally occurring in all domains of life.^47^ Modifications that are present *in vivo* on tRNAs from thermophilic organisms have been shown to increase the thermostability of the RNA. For example, in tRNA from *Pyrodictium occultum*, a hyperthermophile archae on the unmodified, *in vitro*-synthesized tRNA has a melting temperature of 80 °C, while the native modified tRNA has a melting temperature of 100 °C, suggesting that modifications significantly increase the thermostability of tRNA. It is possible that if native modifications were on the thermophilic hammerhead ribozyme, it would continue to be folded and active above 80 °C and would continue to increase in activity. In the context of the full-length hammerhead ribozyme, which has extensive long-range tertiary interactions that are key for aligning helices and C17 for in-line attack,^27^ it is possible that the thermophilic ribozyme could reach a faster rate of catalysis or a rate similar to the mesophilic ribozyme. The minimal mesophilic ribozyme functions well wit a limited tertiary structure, compared to the thermophilic ribozyme, likely because the mesophilic ribozyme is more flexible and able to initiate in-line attack by C17.

The cellular environment is highly complex, and several studies have shown that RNAs fold and behave differently *in vivo* than *in vitro* and that in solutions that mimic the cellular environment, RNAs fold more cooperatively and increase in function.^15,33^ In cells, there are a variety of factors that contribute to native structure formation besides the concentration of free divalent ions, including bound ions, molecular crowding, and metabolites. Recently, the Bevilacqua lab has published several studies showing that Mg^2+^ coordinated to metabolites can still interact with RNA and affect its stability, suggesting that there is much more Mg^2+^ available to coordinate RNA in cells than previously considered.^48–51^ It is possible that in the cellular environment, the thermophilic ribozyme would reach the same level of activity as the mesophilic ribozyme.

Our studies show that increasing GC content in regions of secondary structure is a plausible mode for high-temperature adaptation in ribozymes from thermophiles. Increasing GC content in the stem regions of the minimal hammerhead ribozyme increases the thermostability of the ribozyme at cellular levels of Mg^2+^, while maintaining ribozyme activity, and does so through a simple adaptation of strengthening base pairing. However, this thermal stability comes with a slight functional penalty, resulting in a ribozyme that is active but that self-cleaves at a slower rate than its mesophilic homologue. However, the thermophilic RNA folds in a more cooperative manner than the mesophilic ribozyme, and this beneficial gain in folding cooperativity could compensate for the slower rate of cleavage.

## Supplementary Material



## Notes

Supplemental Figures S1–S3 depicting predicted ribozyme structures from the software package RNAStructure, kinetic gels showing the degradation of the ribozymes at high temperatures, and 2-state fits of the melt experiments (PDF)
