# Supplementary material for: Thermophilic Adaptation of a Minimal Hammerhead Ribozyme Comes with a Functional Penalty
Source: Biochemistry. 2026 Oct 19;65(21):3409–18. doi: 10.1021/acs.biochem.6c00551 (PMC13738344; doi:10.1021/acs.biochem.6c00551)
Supplement: Supplementary file 1 [file bi6c00551_si_001.pdf]

Supporting Information:

Thermophilic Adaptation of a Minimal Hammerhead Ribozyme Comes with a Functional Penalty

Ahmed H. Zegher[1], Samina F. Hanif[1], Evan M. Kielbasinski[1], Claire R. Patterson[2], Carrie Baker[2], Rigel P. Hall[2], Kathleen A. Leamy*[1,2]

[1]Department of Chemistry and Biochemistry, Siena University, Loudonville, NY, 12211

[2]Department of Chemistry and Biochemistry, Gonzaga University, Spokane, WA, 99258

*Corresponding author email: kleamy@siena.edu

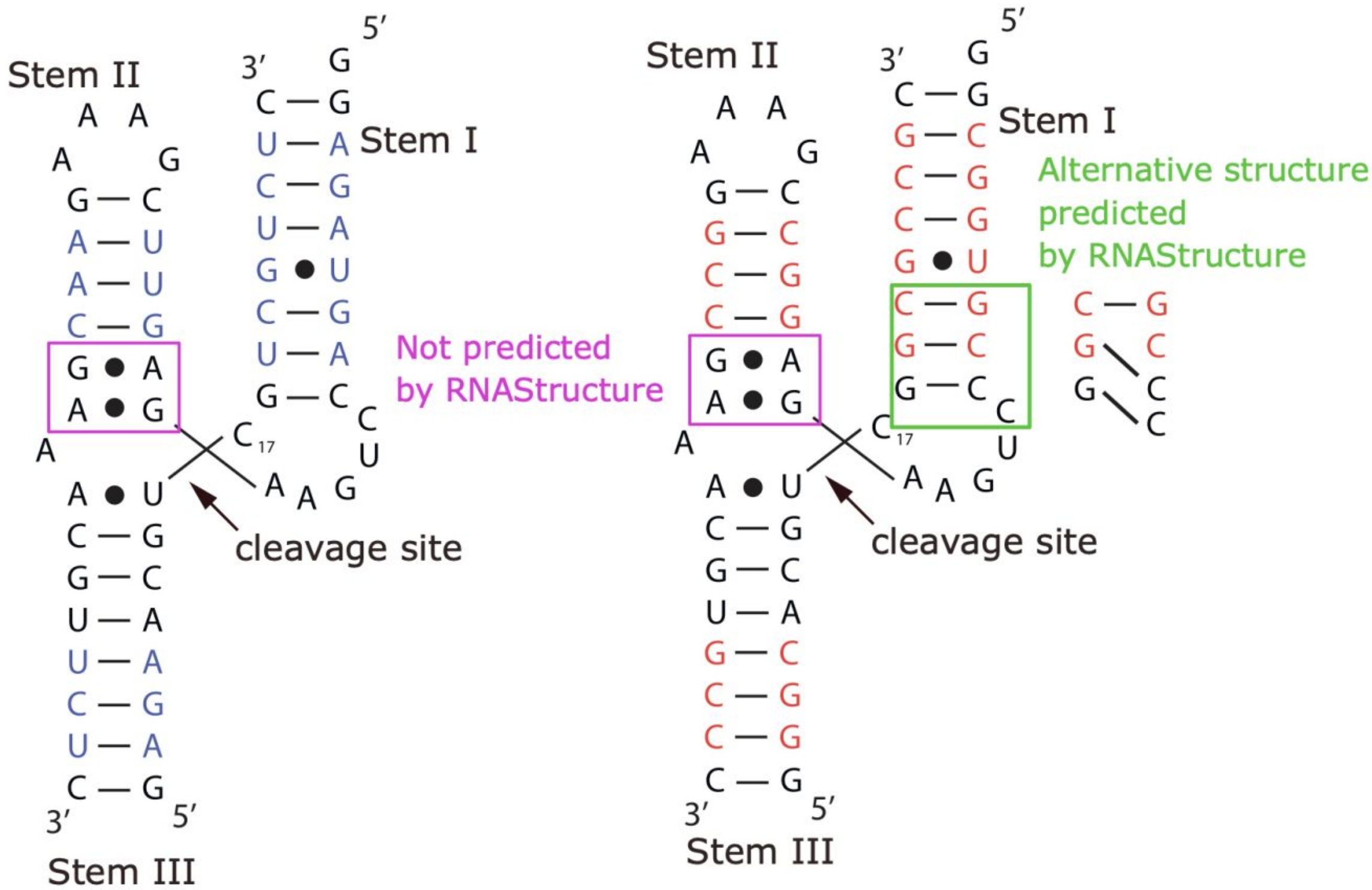

Figure S1. Structures of the **(left)** mesophilic ribozyme and **(right)** thermophilic ribozyme predicted by the software package RNAStructure. Differences between the predicted structure and the expected ribozyme structure are shown in the green box. Two GA mismatches in stem II were not predicted by RNAStructure (purple box) in both ribozymes. Two slightly different structures were predicted from the thermophilic ribozyme that differ in base pairing in stem I (green box). The cleavage site is indicated with an arrow.

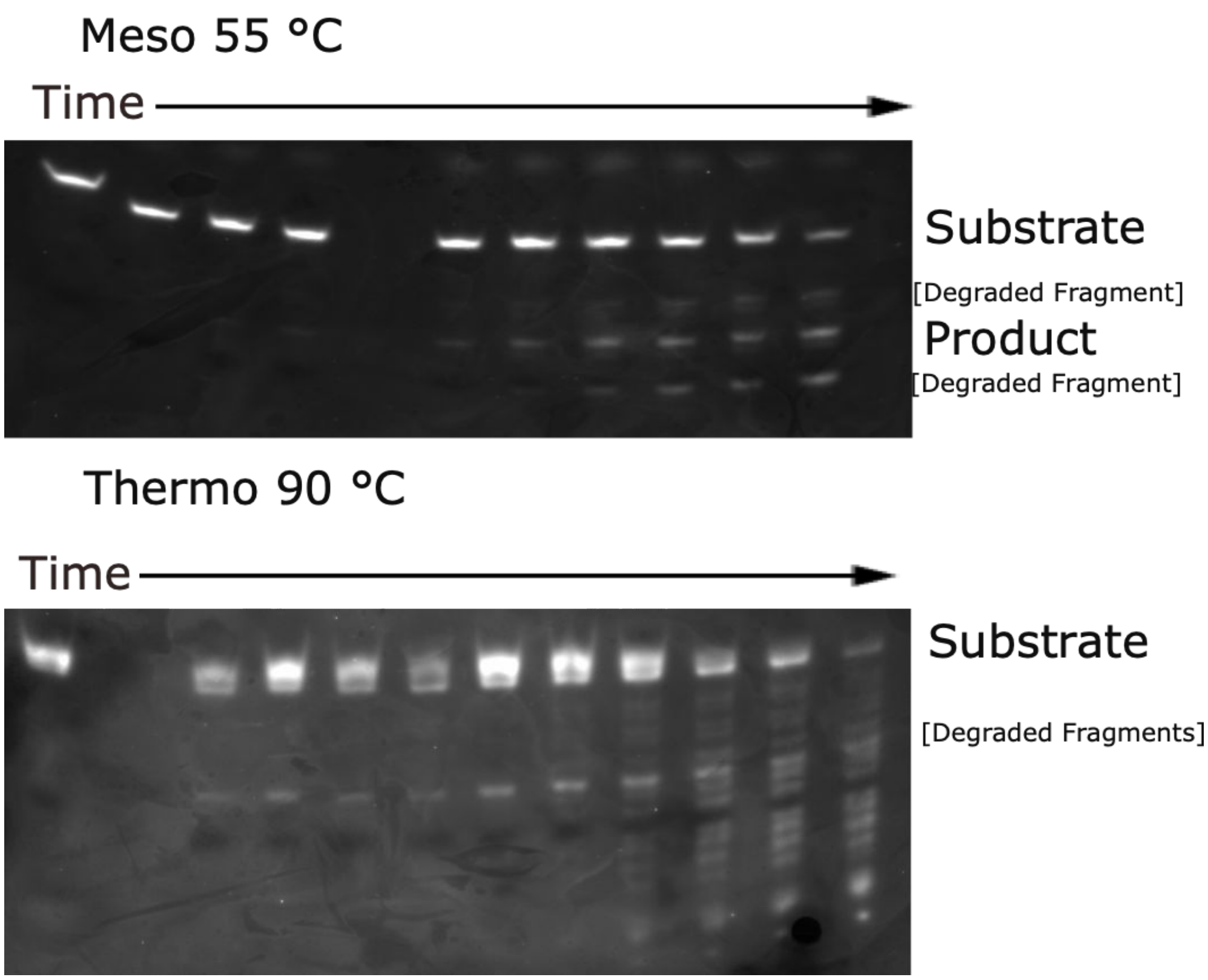

Figure S2. Kinetics gels showing the degradation of the **(top)** mesophilic ribozyme at 55 °C at 2.0 mM Mg^{2+} and **(bottom)** the thermophilic ribozyme at 90 °C in 10.0 mM Mg^{2+} over the reaction time course. Degradation is marked by bands on the gel that are not the substrate or product of the reaction.

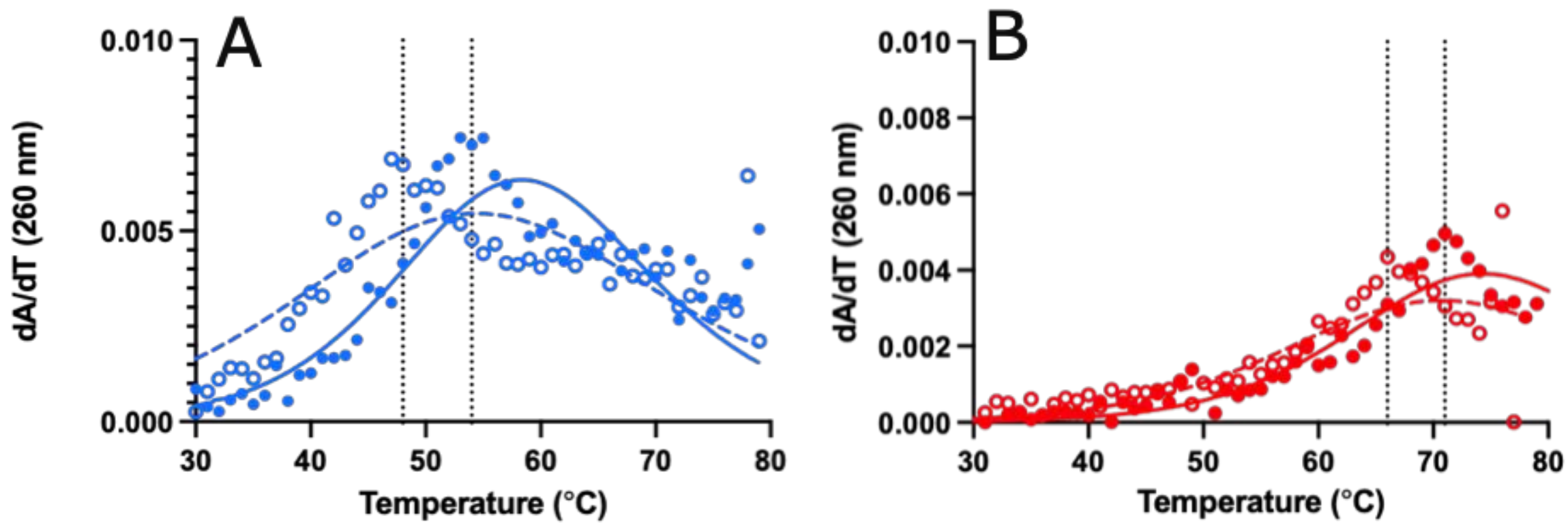

Figure S3. Melt fits of the **(A)** mesophilic (blue) and **(B)** thermophilic (red) ribozymes to a 2-state model (lines) to the raw absorbance data in 2.0 mM Mg^{2+} (open circles, dashed line) and 10.0 mM Mg^{2+} (closed circles, continuous line).
